# Improved vanillin production in baker's yeast through *in silico *design

**DOI:** 10.1186/1475-2859-9-84

**Published:** 2010-11-08

**Authors:** Ana Rita Brochado, Claudia Matos, Birger L Møller, Jørgen Hansen, Uffe H Mortensen, Kiran Raosaheb Patil

**Affiliations:** 1 Center for Microbial Biotechnology, Technical University of Denmark, DK - 2800 Kgs. Lyngby, Denmark; 2 Plant Biochemistry Laboratory, Department of Plant Biology and Biotechnology, University of Copenhagen, DK - 1871 Frederiksberg C, Denmark; 3 Evolva Biotech A/S, Copenhagen, DK - 1870 Frederiksberg C, Denmark; 4 Structural and Computational Biology Unit, European Molecular Biology Laboratory, Meyerhofstrasse 1, 69117, Heidelberg, Germany

## Abstract

**Background:**

Vanillin is one of the most widely used flavouring agents, originally obtained from cured seed pods of the vanilla orchid *Vanilla planifolia*. Currently vanillin is mostly produced *via *chemical synthesis. A *de novo *synthetic pathway for heterologous vanillin production from glucose has recently been implemented in baker's yeast, *Saccharamyces cerevisiae*. In this study we aimed at engineering this vanillin cell factory towards improved productivity and thereby at developing an attractive alternative to chemical synthesis.

**Results:**

Expression of a glycosyltransferase from *Arabidopsis thaliana *in the vanillin producing *S. cerevisiae *strain served to decrease product toxicity. An *in silico *metabolic engineering strategy of this vanillin glucoside producing strain was designed using a set of stoichiometric modelling tools applied to the yeast genome-scale metabolic network. Two targets (*PDC1 *and *GDH1*) were selected for experimental verification resulting in four engineered strains. Three of the mutants showed up to 1.5 fold higher vanillin β-D-glucoside yield in batch mode, while continuous culture of the *Δpdc1 *mutant showed a 2-fold productivity improvement. This mutant presented a 5-fold improvement in free vanillin production compared to the previous work on *de novo *vanillin biosynthesis in baker's yeast.

**Conclusion:**

Use of constraints corresponding to different physiological states was found to greatly influence the target predictions given minimization of metabolic adjustment (MOMA) as biological objective function. *In vivo *verification of the targets, selected based on their predicted metabolic adjustment, successfully led to overproducing strains. Overall, we propose and demonstrate a framework for *in silico *design and target selection for improving microbial cell factories.

## Background

**Figure 1:**
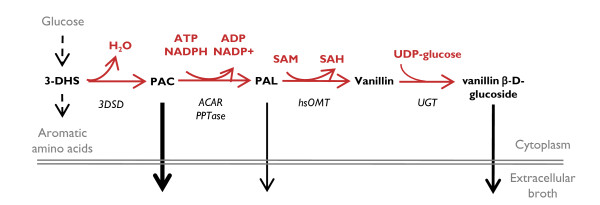
**Schematic representation of the *de novo *VG biosynthetic pathway in *S. Cerevisisae ***(as designed by Hansen *et al *[5]). Metabolites are shown in black, enzymes are shown in black and in italic, cofactors and additional precursors are shown in red. Reactions catalyzed by heterologously introduced enzymes are shown in red. Reactions converting glucose to aromatic amino acids are represented by dashed black arrows. Metabolite secretion is represented by solid black arrows where relative thickness corresponds to relative extracellular accumulation. 3-DSH stands for 3-dedhydroshikimate, PAC stands for protocathechuic acid, PAL stands for protocatechuic aldehyde, SAM stands for S-adenosylmethionine. *3DSD *stands for 3-dedhydroshikimate dehydratase, *ACAR *stands for aryl carboxylic acid reductase, *PPTase *stands for phosphopantetheine transferase, *hsOMT *stands for *O*-methyltransferase, and *UGT *stands for UDP-glycosyltransferase. Adapted from Hansen *et al. *[5].

Vanillin is a plant secondary metabolite and the main constituent of natural vanilla - one of the most important flavouring agents. The annual market for vanillin exceeds 16,000 tons, although only 0.25% of this originates from cured seed pods of the vanilla orchid, *Vanilla planifolia*. The remaining demand for vanillin is fulfilled by chemical synthesis from lignin or fossil hydrocarbons, in particular guaiacol [1]. Sustainable and environmental friendly routes have been proposed for obtaining vanillin through bioconversion of eugenol and ferulic acid by bacteria or fungi [2-4]. To this end, an attractive option was recently reported by *Hansen et al *(2009), who demonstrated *de novo *vanillin biosynthesis from glucose in baker's and fission yeasts as a major step towards developing an environmental friendly and economically sustainable process [5]. The native metabolic precursor for this *de novo *pathway is 3-dehydroshikimate (3-DHS), an intermediate of the shikimate pathway for aromatic amino acids biosynthesis. To convert 3-dehrydroshikimate into vanillin, four genes encoding the required four enzymatic activities were obtained from different organisms, *Podospora pausiceta*, *Nocardia sp*., *Escherichia coli *and *Homo sapiens *(Figure 1) [5]. Inspired by the fact that metabolic engineering has been successfully applied to improve the yield of e.g. sesquiterpenes [6], ethanol [7,8], artemisinic acid [9] and succinic acid [10] production in *Saccharomyces cerevisiae*, we hypothesized that vanillin production could also be improved by implementing a metabolic engineering strategy [11]. An immense collection of systems biology tools, in addition to well-established technologies for genetic manipulation, renders *S. cerevisiae *a very amenable organism for metabolic engineering [12-14].

The aim of this study was to design and construct an improved S. *cerevisiae *vanillin cell factory guided by genome-scale metabolic modelling. *In silico *metabolic engineering algorithms were used to identify target reactions in the metabolic network, knockout of which would lead to improved vanillin production. A set of knockouts that maximizes the flux towards a desired metabolite must be searched for, while the overall flux distribution is determined by the cellular objective function (e.g. maximization of biomass yield). This problem was formulated by Burgard *et al. *(2003) as a bi-level optimization algorithm termed OptKnock [15]. OptKnock can be applied in case of a linear design objective, such as maximizing flux towards a desired metabolite [15]. Optimization of non-linear objective functions, such as productivity, is also of great interest for a variety of metabolic engineering problems. OptGene, an extension of OptKnock, allows maximization of non-linear objective functions, while at the same time accounting for non-linear constraints on the metabolic network [16].

The most widely used approach for calculating flux distribution is flux balance analysis (FBA), where a given flux (or a linear combination of chosen fluxes) is used as the objective function [17]. For microorganisms, biomass maximization is generally accepted as a cellular objective function when simulating flux distributions [18,19]. Alternative fluxes have been proposed as biologically meaningful objective functions, such as maximization of ATP yield [20]. FBA has been successfully applied to predict gene essentiality [21,22], end point of adaptive evolution experiments [23] and optimal metabolic states under given environmental conditions [19]. However, a mutant strain that is not subjected to evolutionary pressure might have a disturbed metabolic network and the principle of optimality for growth may not be prevailing. To address this question, the algorithm Minimization Of Metabolic Adjustments (MOMA) has been suggested by Segrè *et al. *(2002), where it is advocated that the cellular objective for a mutant strain is to minimize its metabolic distance from the wild type flux distribution [24]. In MOMA approach, it is crucial to have a physiologically meaningful wild type flux distribution, as it will strongly influence the predicted phenotype [24]. Within this study, the *S. cerevisiae *genome-scale stoichiometric model iFF708 [22] was used to identify and select target reactions by using OptGene [16]. MOMA [24] was used as the biological objective function with wild type flux distributions spanning three major modes of yeast metabolic physiology. The model-based metabolic engineering strategy was tested experimentally by strain construction and characterization. These research efforts resulted in three mutant yeast strains with significantly increased vanillin production.

## Results and Discussion

### Vanillin β-D-glucoside production in *S. cerevisiae*

Vanillin is toxic to many living organisms. In case of *S. cerevisiae*, growth defect is significant with concentrations as low as 0.5 g/l [5]. Tackling the problem of vanillin toxicity is therefore an important pre-requisite for building an economically viable vanillin cell factory. An elegant solution is glycosylation of vanillin, which is observed in the natural producer *Vanilla planifolia *[25,26]. This strategy was successfully implemented by Hansen *et al. *(2002) in *Schizosaccharmyces pombe *[5]. The glycosylation step implies reduction in the maximum theoretical yield, from 0.57 mmol_Van_/mol_glc _to 0.35 mmol_Van_/mol_glc_. On a mass basis, the maximum achievable yield of 486 mg_van_/g_glc _changes to a relatively modest 293 mg_Van_/g_glc_. Nevertheless, given the toxicity and low solubility of vanillin, reaching high titers is not a favourable option. In case of Vanillin β-D-glucoside (or VG), extracellular concentration up to 25 g/l has been shown not to affect growth and is thereby more suitable for commercial production.

In *S. pombe*, heterologous expression of a gene encoding a plant family 1 glycosyltransferase from *Arabidopsis thaliana *(*UGT72E2) *resulted in 80% conversion of vanillin to vanillin β-D-glucoside [5]. Within this study, *UGT72E2 *was expressed in the vanillin producing *S. cerevisiae *strain VAN286, obtained from Hansen *et al*. [5]. The resulting strain, VG0, was grown in minimal medium containing 20 g/l glucose for 90 h. At this point, the culture was harvested and the extracellular broth was analysed for the presence of vanillin and VG. VG had significantly accumulated in the broth (>100 mg/l), while vanillin was barely detectable (<7 mg/l) indicating efficient conversion of vanillin into VG.

### *In silico *design

Existence of a large number of alternative flux routes (pathways) in genome-scale metabolic models requires the use of experimental constraints in order to obtain physiologically meaningful flux distributions. This is even more so in the case of MOMA, where predictions of mutant flux distributions will be highly dependent on the solution provided for the wild type (or reference) flux distribution [24]. Thus, the model formulation should be capable of taking into account the basic metabolic physiological characteristics of the strains under consideration. In particular, when grown with a fermentable carbon source, *S. cerevisiae *has the ability to grow in the absence of oxygen, producing ethanol as a major by-product. In the presence of oxygen, respiration occurs but if the glucose concentration and/or uptake rate surpasses a critical threshold value, the metabolism becomes a combination of respiration and alcoholic fermentation [27,28].

#### Reference metabolic states

Different metabolic states of *S. cerevisiae *are characterized by different nutrient uptake rates, different metabolite production rates and different biomass yields. Consequently, it is important to decide which metabolic state/s should be used to constrain the metabolic model in order to obtain reliable target predictions for genetic manipulation towards improved productivity. In addition, it is to be expected that different constraints (corresponding to different metabolic states) may lead to different suggestions for the metabolic engineering targets. To address this issue, we used three different scenarios for obtaining the reference flux distribution for MOMA simulations. Reference 1 represented exclusive respiratory metabolism, characterized by no ethanol formation and low glucose uptake rate. Reference 2 was simulated for respiro-fermentative metabolism, characterized by high glucose uptake rates, alcoholic fermentation and active respiration. Since both flux distributions were obtained by using FBA for maximizing biomass production, no accumulation of VG or related compounds was predicted. A third FBA simulation was performed to obtain the flux distribution for VG0 (Reference 3). The model was constrained using data obtained in this study from chemostat cultivations at a dilution rate of 0.1 h^-1^. Highly fermentative metabolism was observed at this dilution rate indicating limited respiratory capacity of the strain. Together, the three different reference flux distributions span all relevant life styles of *S. cerevisiae*, and may therefore be used to identify potential targets for genetic manipulations by using OptGene simulation framework. As steady state approaches cannot predict changes in substrate uptake rates, and hence productivity for mutants, Patil *et al*. (2005) suggested the use of Biomass Product Coupled Yield (BPCY) for design objective. BPCY is defined as multiplication of product yield and biomass flux [16].

#### Assessment of in silico predictions

**Figure 2:**
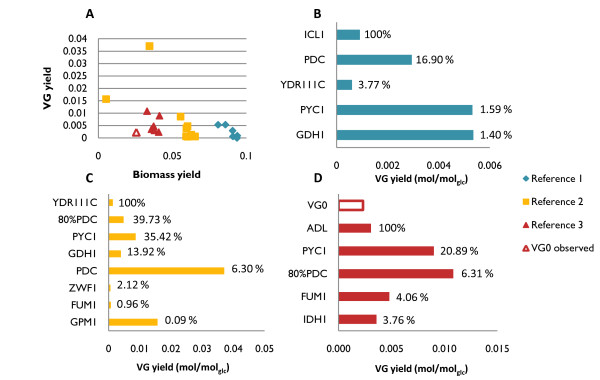
**Comparison of targets predicted by OptGene for improved VG productivity**. **A **- Biomass versus VG yield is represented for each knockout mutant phenotype obtained after OptGene simulation using three different reference flux distributions for MOMA. Experimental yields observed for VG0 are represented by the red empty triangle and bar. **B/C/D **- The predicted VG yield (mol/mol_glc_) obtained for each knockout mutant after OptGene simulation using Reference 1/2/3 is given by the length of the coloured bars. For each reference flux distribution, the R^3 ^score was estimated for each of the mutants was calculated and normalized to the mutant presenting the highest value: MA_mut_/MA_max_*100. 100% represents the mutant with highest R^3 ^score for a given flux distribution. Candidate 80%PDC is not a knockout *in silico *mutant, rather its PDC reaction is constrained to 80% of the upper bound.

Simulations were performed using OptGene [16] for predicting up to six reaction knockout targets. Improved VG production was not predicted when using maximization of biomass production as biological objective, while the use of MOMA [24] suggested interesting targets even after a single reaction deletion. The targets suggested by OptGene were verified for optimality by using OptKnock [15]. Among a variety of possible target reactions (Figure 2A), biomass and VG yield are generally related with an inverted trend, so when predicted biomass yield is high, predicted product yield tends to be modest and vice-versa. Target selection for experimental validation must strike a good balance between improved VG production and a reasonable prediction for biomass yield (Figure 2A). Metabolic adjustment (as defined by Segrè *et al.*, 2002 [24]) was used as an additional factor to rank each of the candidate target sets. Briefly, metabolic adjustment of a mutant is defined as the Euclidean distance between the reference and the mutant flux distribution vectors. Our hypothesis is that smaller metabolic adjustments are more likely to be achieved *in vivo *than large adjustments. This was taken into account by introducing a new metric for ranking *in silico *predicted mutants, *viz.*, the Reward-Risk-Ratio (R^3^), defined as the ratio between BPCY (reward) and metabolic adjustment (risk). The most interesting targets obtained following ranking of each prediction according to the R^3 ^score are presented in Figure 2 (B, C and D).

Depending on the reference flux distribution used in MOMA, different targets were suggested for knockout. Some of the targets, for example GDH1, were identified in more than one physiological growth condition. However, their positions following the R^3 ^ranking are very different, demonstrating that the best targets in one physiological scenario are not necessarily the best targets in another. The results of the prediction analysis imply that VG production is favoured at those physiological conditions which lead to respiro-fermentative metabolism as compared to physiological conditions resulting exclusively in respiratory metabolism. The outcome of minimal metabolic adjustment is to divert the ethanol flux towards formation of VG rather than diverting the flux from biomass constituents. Indeed, several of the candidates suggested in Figure 2 are predicted to have lower ethanol formation than the reference. Likewise, the biomass yield predicted for all the suggested mutants (with VG0 flux distribution as a reference) is slightly higher than for the reference, again at the expense of ethanol formation. Among the targets depicted in Figure 2, pyruvate seems to embrace a relevant stoichiometric bottleneck since several reactions from pyruvate metabolism were suggested as targets for deletion while considering different reference flux distributions. Furthermore, genes related to the metabolism of ammonium, the pentose phosphate pathway and central carbon metabolism in general, were also identified.

#### Target selection

The targets for *in vivo *implementation were selected by giving strong emphasis to the R^3 ^score of different targets under different simulation conditions, in combination with a manual evaluation of the suitability of the putative gene targets based on available data in the literature. The literature and database search included the possible existence of iso-enzymes, experimentally observed single gene deletion phenotypes and the assessment of the importance of regulatory links to other processes [29]. Based on this analysis, two gene candidates, *PDC1 *(Pyruvate decarboxylase) and *GDH1 *(Glutamate dehydrogenase), were selected as the candidates for strain construction and characterization. The Gdh1 catalyzed reaction was predicted as a prime target for knockout when using respiratory or respiro-fermentative metabolism, and in both cases, relatively large metabolic adjustment was predicted. *GDH1 *encodes an NADPH-dependent glutamate dehydrogenase involved in ammonium metabolism through glutamate biosynthesis, which is reported to provide 85% of the cellular nitrogen sources [30]. Ammonium metabolism has been extensively studied in *S. cerevisiae *and in particular, deletion of *GDH1 *was previously used as a metabolic engineering strategy for improving ethanol and sesquiterpenes production [6,8,31]. The engineered strains were reported to have an increased NADPH pool, a kinetic/thermodynamic feature which was not considered in our modelling strategy. Therefore, even though the deletion of this gene was not suggested when the network was constrained with experimental results (VG0), we expect the deletion of this gene to thermodynamically favour the conversion of PAC to PAL by ACAR.

Pyruvate is a key metabolite in *S. cerevisiae *metabolism and the branch point between respiratory and fermentative metabolism. Pyruvate decarboxylases (PDCs) have a crucial significance for fermentation, since this decarboxylation reaction converts pyruvate to acetaldehyde, an intermediate towards ethanol formation [32]. The *S. cerevisiae *genome harbours three PDC structural genes (*PDC1, 5 *and *6*), one regulatory gene (*PDC2*) and two other genes with potential contribution towards PDC activity (*PDC3 *and *4*) [32,33]. Complete suppression of pyruvate decarboxylase activity (*pdc1*Δ, *pdc5Δ & pdc6Δ*) creates a mutant that is unable to grow on glucose as sole carbon source [33,34]. By *in silico *analysis, PDC was found as a target to increase the formation of VG considering both respiratory and respiro-fermentative reference flux distributions for MOMA, but not when VG0 data was used to constrain the network. Furthermore, complete absence of pyruvate decarboxylase activity under a highly fermentative mode was predicted to result in zero growth, as lack of PDC activity would require a very large metabolic adjustment. This observation, in combination with the experimental data available in the literature for the mutant without PDC activity [33,34], raise an interesting question - what would be the effect of only partial reduction of PDC activity, e.g. by deletion of one of the structural genes? In order to simulate this situation, PDC flux was constrained to 80% of the flux observed in the reference strain. This simulation predicted positive growth as well as higher VG production in comparison to other targets such as *GDH1 *(Figure 2). Of the three PDC structural genes, *PDC1 *was selected as a target for deletion *in vivo *as there is experimental evidence that its removal results in ~30% reduction of total pyruvate decarboxylase activity [34].

### Strain construction and characterization

Following the selection of the two target genes, two single gene deletion mutants, *gdh1*Δ and *pdc1Δ*, were constructed in the VG0 background resulting in VG1 and VG2, respectively. To test whether simultaneous deletion of *PDC1 *and *GDH1 *would have a positive synergistic effect on VG accumulation, a mutant with both deletions was obtained (VG3). The strains were initially characterized in batch cultures in 2L well-controlled bioreactors, using minimal medium and an initial glucose concentration of 20 g/l (Table 1). The mutant VG2 (*pdc1Δ*) showed an overall increased fitness compared to the reference strain as documented by a 43% higher maximum specific growth rate (μ_max_, (h^-1^) α doubling time^-1^) in comparison to VG0. Likewise the yield of biomass on glucose (Y_S X_, g_DW_/g) is 40% higher than observed for VG0. On the other hand, the mutant VG1 (*gdh1*Δ) showed reduced strain fitness, illustrated by poor μ_max _and reduced Y_S X_. These adverse effects on VG1 fitness were partially relieved by deletion of *PDC1*, as documented by slightly improved μ_max _and Y_S X _values for the strain VG3 (*pdc1Δgdh1Δ*). The decreased fitness of the strains in which *GDH1 *was deleted, is due to a reduced nitrogen assimilation rate [35]. In the absence of *GDH1*, the GS-GOGAT system (coaction of two enzymes, a glutamate synthase, *GLT1*, and a glutamine synthetase, *GLN1*) [36,37] and the glutamate dehydrogenase, coded by *GDH2 *[38], are responsible for ammonia assimilation. Both alternatives use NADH instead of NADPH, thus explaining the high metabolic adjustment predicted for the *GDH1 in silico *mutants (Figure 2B and 2C). In comparison with Gdh1, lower activity has been reported for both of the alternative systems. Consequently, overexpression of the enzymes involved in the alternative pathways is a required step in order to recover the cellular fitness [39]. We proceeded experimentally with *GDH2 *overexpression as the use of the GS-GOGAT system has the disadvantage of using an important cellular resource - ATP.

**Table 1:** Physiological parameters for the reference and metabolically engineered strains in batch cultivation.

Strains	Engineered Genotype	**μ**_**max**_^**a**^	**Y**_**S X**_^**b**^	**Y**_**S EtOh**_^**c**^	**Y**_**S gly**_^**d**^
VG0		0.14	0.10	0.23	0.05
VG1	*gdh1∆*	0.10	0.07	0.25	0.03
VG2	*pdc1∆*	0.20	0.14	0.23	0.07
VG3	*pdc1 ∆gdh1∆*	0.11	0.10	0.27	0.05
VG4	*pdc1 ∆gdh1∆ *↑*GDH2*	0.17	0.17	0.25	0.07

^a^Maximum specific growth rate, h^-1^.

^b^Overall yield of biomass on substrate, g_DW_.g_glc_^-1^

^c^Overall yield of ethanol on substrate, g_eth_.g_glc_^-1^

^d^Overall yield of glycerol on substrate, g_Gly_.g_glc_^-1^

The biomass overall yield in glucose (Y_S X_) was calculated based on all the biomass obtained after glucose and ethanol consumption. Ethanol and glycerol yields were calculated based on production only in the glucose consumption phase.

The resulting strain VG4 (*pdc1Δgdh1Δ *↑*GDH2*) showed a significantly improved cellular fitness compared to VG3 with μ_max _and Y_S X _values similar to those observed for VG2 and better than those obtained with VG0. At these experimental conditions (batch cultivation), none of the introduced mutations seem to affect ethanol production to any significant extent except that the strains harbouring the *GDH1 *deletion tend to show a slight increase in the substrate specific yield of ethanol.

**Figure 3:**
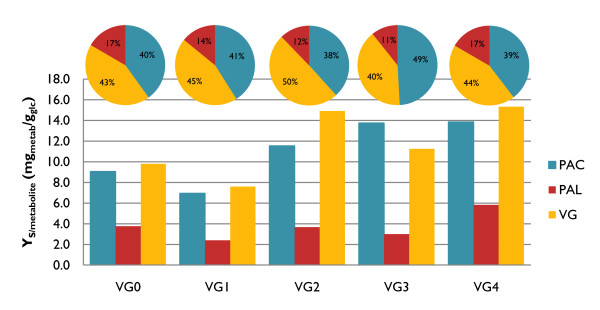
**Vanillin β-D-glucoside yield observed for the reference strain (VG0) and metabolically engineered mutants (VG1-4) in batch cultivations**. Substrate overall yield for vanillin β-D-glucoside (Y_S VG_, mg_VG_/g_glc_), protocatechuic acid (Y_S PAC_, mg_PAC_/g_glc_) and protocatechuic aldehyde (Y_S PAL_, mg_PAL_/g_glc_) obtained for the reference and engineered strains in batch culture. Pie charts are presented to illustrate relative distribution of PAC, PAL and VG for each strain.

Accumulation of several intermediates in the vanillin pathway was observed following growth of all four different mutants. In combination, protocatechuic acid (PAC) and protocatechuic aldehyde (PAL) accounted for more than 50% of the total products formed. Vanillin, other intermediates and by-products such as vanillic acid and vanillyl alcohol were found in very low amounts and were not taken into account for further analysis. The strain VG1 showed the lowest total yield of compounds related to the vanillin biosynthetic pathway (Figure 3), which might be related to the general decreased fitness observed in this strain. The other three engineered strains displayed better performance than VG0 concerning VG production. Single deletion of *PDC1 *in the strain VG2 resulted in an increase of 52% in VG yield and a 30% increase in the overall yield of the compounds from the *de novo *vanillin pathway. Despite the adverse effect of the *GDH1 *deletion, double deletion of *PDC1 *and *GDH1 *in VG3 resulted in 15% increase in VG production compared to VG0. In contrast to strain VG0, the yield of VG increased by over 55% in the strain VG4. In all of the strains, increase in VG was accompanied by accumulation of PAC and PAL in different relative amounts (Figure 3). The most desirable distribution among these metabolites was observed in the case of VG2, where 50% of the total products formed was VG. Overall, the strain engineering carried out during this study led us from a strain producing 9.8 mg_VG_/g_glc _to a strain producing 15.3 mg_VG_/g_glc _in batch cultivation.

The extent to which VG production is affected by the presence or absence of alcoholic fermentation is still unknown. Above a critical threshold for glucose uptake rate, co-existence of respiration and fermentation takes place and the biomass yield is significantly lower due to carbon channelling towards ethanol, known as the Crabtree effect [28]. This phenomenon, also termed overflow metabolism, is typically observed in batch cultivation where the initial glucose concentration is usually above the critical threshold for mixed metabolism. In chemostat cultivation, glucose concentration is kept at very low levels and below the critical dilution rate (strain-specific), the glucose uptake rate is low enough to ensure exclusive respiratory metabolism [27,40,41].

#### Respiratory vs fermentative metabolism for VG production

In an attempt to test whether reduced fermentation would lead to increased biomass and/or VG production, the VG0 reference strain and the best performing mutants in batch cultivations (VG2 and VG4) were selected for further characterization in glucose-limited chemostat cultures at a dilution rate of 0.1 h^-1^and 20 g/l feed glucose concentration. VG2 showed increased fitness as demonstrated by a higher biomass yield (Y_S X_) accompanied by slightly increased ethanol and a slightly decreased glycerol yields, as compared to VG0 (Table 2). Under these conditions, VG2 shows 5.6 mg_VG_/g_glc_, 40% higher VG yield than VG0. In contrast, VG4 displayed a large decrease in Y_S X_, while ethanol and glycerol production were significantly increased (Table 2)., At the same time, the VG production observed for this strain remains very similar to that of the reference strain (Table 2).

**Table 2:** Physiological parameters for the reference and metabolically engineered strains in chemostat cultivation at dilution rate 0.1 h^-1^.

Strain	VG0	VG2	VG4
**Engineered Genotype**		***pdc1∆***	***pdc1 ∆gdh1∆ *↑*GDH2***

Y_S X _(g_DW_.g_glc_^-1^)	0.151 ± 0.008	0.159 ± 0.003	0.115 ± 0.005
Y_S eth _(g_eth_.g_glc_^-1^)	0.283 ± 0.005	0.290 ± 0.008	0.32 ± 0.01
Y_S gly _(mg_gly_.g_glc_^-1^)	3.4 ± 0.3	2.1 ± 0.4	11 ± 9
Y_S acet _(mg_acet_.g_glc_^-1^)	7.3 ± 0.3	7.6 ± 0.3	4 ± 2
Y_S PAC _(mg_PAC_.g_glc_^-1^)	27 ± 2	23.5 ± 0.3	15.5 ± 0.3
Y_S PAL _(mg_PAL_.g_glc_^-1^)	7 ± 2	9 ± 2	7.2 ± 0.2
Y_S VG _(mg_VG_.g_glc_^-1^)	4 ± 1	5.6 ± 1	4.16 ± 0.07

r_s_(mmol_glc_.g_DW_^-1^.h^-1^)	3.9 ± 0.2	3.5 ± 0.3	4.8 ± 0.2
r_eth_(mmol_eth_.g_DW_^-1^.h^-1^)	4.3 ± 0.3	3.9 ± 0.3	6.1 ± 0.4
r_gly_(mmol_gly_.g_DW_^-1^.h^-1^)	0.026 ± 0.003	0.014 ± 0.004	0.10 ± 0.08
r_acet_(mmol_acet_.g_DW_^-1^.h^-1^)	0.086 ± 0.006	0.079 ± 0.006	0.05 ± 0.003
r_PAC_(mmnol_PAC_.g_DW_^-1^.h^-1^)	0.12 ± 0.01	0.10 ± 0.01	0.087 ± 0.03
r_PAL_(mmol_PAL_.g_DW_^-1^.h^-1^)	0.036 ± 0.008	0.040 ± 0.007	0.045 ± 0.003
r_VG_(mmol_VG_.g_DW_^-1^.h^-1^)	0.009 ± 0.003	0.011 ± 0.002	0.012 ± 0.001

Substrate yields for biomass (X), ethanol (eth), glycerol (Gly), atetate (Ace), protocathechuic acid (PAC), protocathechuic adehyde (PAL) and vanillin β-D-glucoside (VG) on glucose (glc) are represented by Y_S X (or metabolite_). Specific glucose uptake rate is represented by r_S_. Specific production rate for ethanol, glycerol, acetate, PAC, PAL and VG are represented by r_metabolite_.

Triplicates of all chemostats were performed.

**Figure 4:**
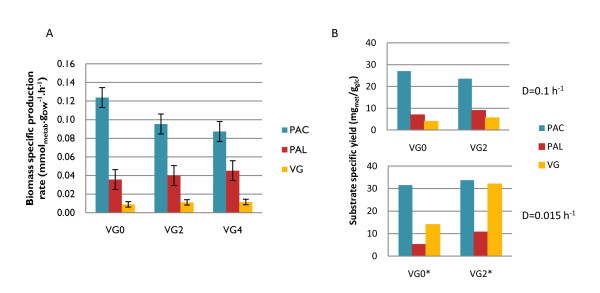
**Vanillin β-D-glucoside yield observed for the reference strain (VG0) and metabolically engineered mutants (VG1-4) in continuous cultivations**. **A **- Biomass specific production rate (mg_metab_.g_dw_^-1^.h^-1^) for protocatechuic acid (PAC), protocatechuic aldehyde (PAL) and VG in glucose limited chemostat cultivation at dilution rate of 0.1 h-^1^. **B - **Substrate specific yield (Y_S Metab_, mg_metab_/g_glc_) for PAC, PAL and VG for strains VG0 and VG2 in glucose limited chemostat cultivation at different dilution rates - 0.1 h-^1^(Top) and 0.015 h-^1^(*, Bottom).

The strain VG0 showed a glucose uptake rate (r_S_) of 3.9 ± 0.2 mmol_glc_g_gw_^-1^.h^-1^, whereas VG2 has an uptake rate of 3.5 ± 0.2 mmol_glc_g_gw_^-1^.h^-1^. The lower glucose uptake decreases the overflow metabolism in the strain, leading to decreased rates of ethanol, glycerol and acetate formation. At the same time, VG2 exhibits a higher conversion of PAC into the products of the later steps in the vanillin pathway (Figure 4A). The strain VG4 displays a lower biomass yield on glucose and a corresponding higher r_S _value suggesting an increased overflow metabolism (Table 2). Accordingly, a significantly increased production of ethanol (1.5-fold) and glycerol (4-fold) was observed. Despite the severe impact of the overflow metabolism on the biomass yield of the VG4 strain in the conditions tested, better conversion of PAC into downstream metabolites was observed, especially to PAL (Figure 4A). A possible interpretation of this result is that the VG4 strain has higher availability of NADPH, due to the engineered reduced demand for this cofactor for ammonium metabolism. This most likely affords a thermodynamically more favoured conversion of PAC into PAL, as hypothesised during target selection.

The predominantly respiro-fermentative metabolism observed for all engineered strains (VG0, VG2 and VG4) implies that the critical dilution rate (indicative of respiratory capacity) of the strains is very low. By gradually decreasing the dilution rate in a glucose-limited chemostat, we verified that the critical dilution rate for both VG2 and VG0 was below 0.015. Such a low respiratory capacity could be the result of combined effects of product/by-products toxicity and due to the background of the strain. Employing this experimental setup made it possible to lower the ethanol production rate to 0.130 mmol_eth_g_gw_^-1^.h^-1 ^for VG0 and to 0.065 mmol_eth_g_gw_^-1^.h^-1 ^for VG2, while the rate of glucose uptake was 0.37 and 0.30 mmol_glc_g_gw_^-1^.h^-1^, respectively. With VG2, the VG concentration in the broth was 500 mg/l, (32 mg_VG_/g_glc_), a two-fold increase compared to that of VG0, which produced 250 mg/l (15 mg_VG_/g_glc_) (Figure 4B). These results confirm that VG2 has better respiratory capacity than that of the reference strain VG0 and that lowering the overflow metabolism results in higher VG yield. The fact that higher yields of the intermediates PAC and PAL as well as of the final product, VG, are obtained at low dilution rates, suggests a significantly increased flux through the vanillin biosynthetic pathway. In both strains, the observed conversion of PAL into VG is more efficient at low dilution rates. This confirms that low overflow metabolism is linked to an increased precursor and/or cofactors supply, enabling higher VG productivity.

### Analysis of the experimental results

In an attempt to better understand the metabolic flux changes at the whole network level and, therefore, the basis for the observed improved VG production, the flux phenotypes of VG0, VG2 and VG4 were simulated by using FBA [17]. The experimental results obtained from the chemostat cultures, i.e. glucose uptake rate and biomass yield, ethanol, acetate, glycerol and CO_2 _production rates, were added as constraints to the metabolic model. The bounds for the deleted genes were set to zero, as in the case of *GDH1 *deletion in VG4. However the choice of constraints for the *PDC1 *deletion is not straightforward, as deleting *PDC1 *does not mean that pyruvate decarboxylase activity will be zero. To circumvent this issue, and to account for different glucose uptake rates of the mutants, the upper bound for PDC flux was identified in each condition. Subsequently, the phenotypes were simulated with upper bound for PDC constrained to 80% of the previously found upper bound.

#### Flux variability analysis

**Figure 5:**
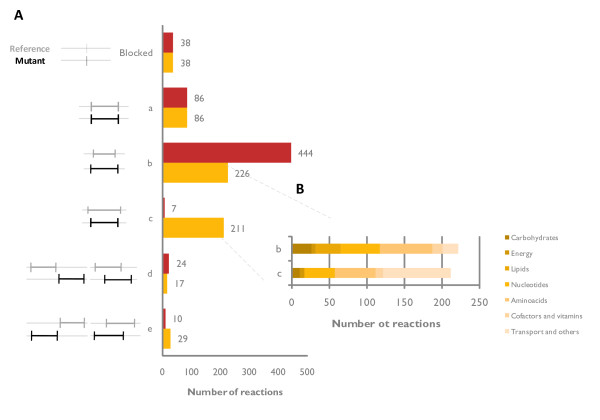
**Flux variability analysis**. Reactions were classified based on the comparison of their flux variability range between the reference VG0 and the mutants VG2 and VG4. **A**- The scheme on the left-hand side illustrates the flux variability ranges defining the six different categories (Blocked and *a *to *e*). Flux variability range for the reference strain (VG0) is represented in gray, and the mutant in black. The distribution of VG2 and VG4 reactios among different categories are presented in the bar chart, yellow and red, respectively. **B**- The reactions from mutant VG2 belonging to categories *b *and *c *are further classified accordingly to their metabolic function.

The flux distributions obtained with FBA are guaranteed to be optimal, but not necessarily unique due to the existence of a large number of alternative routes. This renders the transformation of the experimentally determined levels of the products obtained into intracellular fluxes, a difficult task. Nevertheless, stoichiometric simulations provide an estimate of the possible range of flux values for every reaction in the network. Fluxes which are unique will have the same maximum and minimum possible values. The flux ranges of all reactions of VG2 and VG4 were calculated and compared with those of VG0, resulting in different categories as illustrated in Figure 5. The first category consists of those reactions for which flux is infeasible at steady-state, i.e. blocked reactions. Among the remaining reactions (~570), only 50 have unique flux values for the reference strain and for the mutants (VG2 and VG4). Almost all of these reactions belong to category *a*, i.e. with no change between the VG0 reference strain and the tested mutants. The chemostat experiments were carried out at the same dilution rate for all the strains, and as a consequence, the simulation of reactions directly coupled to biomass biosynthesis all fall within this category. Examples include reactions from lipid, nucleotide and amino acid metabolism.

The categories *b *and *c *contain the majority of the reactions (~70%) for the VG2 and VG4 strains, yet the distribution among the two categories is not alike for the two mutant strains. In the case of VG2, 226 reactions exhibit a larger range than observed for the reference strain (category *b*) and a similar number of reactions exhibit a smaller range than observed in the VG0 (category *c*). For the VG4 strain, most of the reactions fall in category *b*, as expected due to its higher glucose uptake rate. The VG2 strain exhibits a decreased glucose uptake rate and the same biomass formation rate as compared with VG0. Nevertheless, several reactions from the central carbon metabolism show an increased flux range (category *b*). These reactions mainly belong to the tricarboxylic acid (TCA) cycle, the pentose phosphate pathway and gluconeogenesis, reflecting that this strain has decreased overflow metabolism and ethanol production. A more active respiration in this strain is further confirmed by the *in silico *predicted increased oxygen uptake rate. Even though this reaction is found in category *c *(with decreased flux range), its lower bound is higher in VG2 than in VG0.

The remaining reactions present flux variability ranges with partial or no overlap between the reference strain and the mutants. These reactions were grouped in category *d*, where the mutant flux upper bound is higher than the reference flux upper bound, and category *e *where the mutant flux lower bound is lower than the reference flux lower bound. Together, categories *d *and *e *comprise the reactions with the clearest differences between the mutants and the reference. These include reactions related to product formation (e.g. biosynthesis of S-adenosylmethionine - the methyl-group donor in the vanillin pathway) and glucose uptake (which was experimentally determined), as well as reactions from the ammonia metabolism.

Despite the increased VG production, the VG2 strain exhibits a decreased flux through the aromatic amino acid biosynthesis pathway from which the vanillin precursor is derived. Likewise, this strain shows a reduced total flux through the VG pathway. The same trend was found for the VG4 strain, implying that the metabolic network is being adjusted for increased PAL and VG production at the expense of a reduction of the total carbon flow into aromatic amino acids until the VG biosynthesis branch. In actual fact, the reaction after which the production is increased is the conversion from PAC to PAL. This reaction uses NADPH and ATP, two of the most highly connected metabolites and cofactors that are competed for by growth requirement.

#### Metabolite-centric analysis

To systematically explore the usage of cofactors and other metabolites in the engineered strains, the turnover of these metabolites can be calculated by summing all the fluxes which are producing (or consuming) them [42]. As the unique values of all the fluxes are unknown due to alternative optima inherent to FBA of metabolic networks, the minimum metabolite turnover was calculated by solving a linear programming problem (LPP) for the *minimization of metabolite turnover *(see materials and methods for details). This LPP formulation guarantees to find the minimum turnover of a given metabolite that ensures the observed phenotype. The direction of optimization, *i.e. *minimization, not only avoids the unbound cyclic fluxes around the metabolite under question, but also confers with the biological hypothesis of minimal resource allocation by the cell in terms of enzyme expression. Minimum metabolite turnover denotes how much flux needs to pass through a given metabolite, although the distribution of this flux among possible reactions may not be unique in all cases. Nevertheless, the turnover calculated in this way provides a lower bound on the flux through a metabolite that can be used in complementation with flux variability analysis.

**Figure 6:**
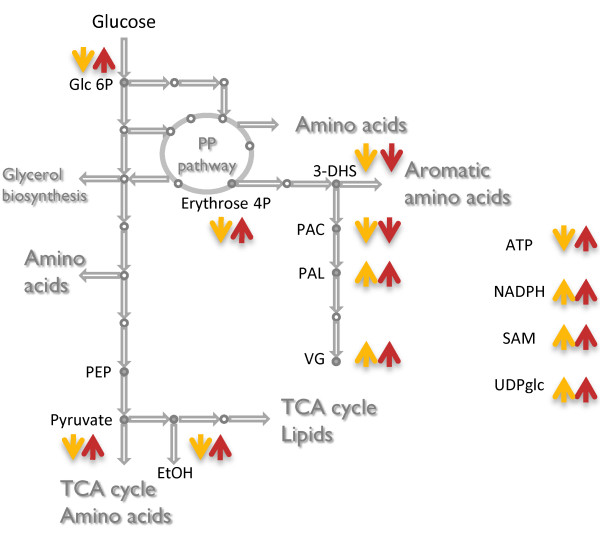
**Minimum turnover of selected metabolites from the central carbon metabolism and from the VG biosynthetic pathway **(including cofactors). Metabolites from the central carbon metabolism: glucose-6-phosphate, erythrose-4-phosphate, pyruvate and ethanol; Metabolites from the VG biosynthetic pathway (ATP, NADPH, SAM and UDP-glucose). Metabolites for which minimum turnover was calculated are represented by filled circles, metabolites for which no minimum turnover was calculated are represented by open rings. Reactions are represented as arrows. Qualitative variation of the minimum turnovers relatively to the reference (VG0) is shown by the arrows next to each metabolite; yellow corresponds to VG2 while red corresponds to VG4.

Besides NADPH and ATP, the minimum turnover of some other relevant metabolites was also calculated (Figure 6). The minimum turnover for PAC is lower in the strains VG2 and VG4 than for VG0; while for PAL and VG the opposite trend is verified, in agreement with the flux variability analysis. An increase in the glucose uptake rate will result in an increase in glycolysis and pentose phosphate pathway, which is reflected in the increased minimum turnover of pyruvate and erythrose-4-phosphate in the VG4 strain. On the other hand, the VG2 strain exhibits a decreased glucose uptake rate and consequently less flux through glycolysis and pentose phosphate pathway. The same trends apply to ATP, implying that most ATP available in the cell is being produced from glycolysis. NADPH, S-adenosylmethionine and UDP-glucose minimum turnovers are increased for both simulated phenotypes, reflecting the increased flux through ACAR, hsOMT and UGT leading to PAL and VG. The analysis above provides insight into the intracellular flux changes and pinpoints metabolites that play a role in the engineered strains.

Three VG overproducing strains were successfully designed and constructed during this study. Systems biology tools, such as the yeast genome-scale model, were used throughout the study, from the strain design to the analysis of the physiological data resulting from the fermentation studies of the constructed mutants. The *in silico *predicted increase in the product yield was above 2-fold (Figure 2A). Indeed, 2-fold improvement in the yield was observed for one of the mutants, albeit at low dilution rate. On the other hand, the observed improvement in batch cultivation was close to a 1.5-fold increase. The limited kinetic and regulatory information, as well as the lack of tools to integrate such information within genome-scale metabolic models, are the main likely reasons for the discrepancy between the predicted and the experimentally determined yields. The need for accounting of the regulatory information is even more apparent when considering the fact that an isoenzyme was chosen as a target. Effects of deletion of a gene coding for an isoenzyme on the flux re-routing are hard to predict. In reality, quantitative prediction of flux distributions following down-regulation or overexpression of a gene (or corresponding enzyme activity) is still in its infancy. Advances within this area would require more experimental data on the regulation of metabolic networks as well as flux simulation tools that can integrate such information. Regulation at both the hierarchical and metabolic level is of particular relevance for vanillin production as the shikimate pathway for aromatic amino acids biosynthesis is tightly regulated in yeast [43]. As an example, Luttik and co-workers were able to increase the total flux through this pathway by 4.5-fold in *S. cerevisiae *through alleviation of the DAHP synthase feed-back inhibition mechanisms [44].

## Conclusions and Future Perspectives

The *in silico *strategy design revealed the sensitivity of the target predictions towards the reference flux distribution used for simulating the mutants. To this end, it was crucial to use basic physiological knowledge for simulating different relevant yeast phenotypes. On the experimental front, three yeast strains with improved vanillin β-D-glucoside production were designed based on a model-guided metabolic engineering strategy. Physiological characterization of these mutants in chemostat cultivation (and subsequent *in silico *flux analysis) allowed us to conclude that they display increased cofactor availability for VG production. Further increase of cofactor availability would be of great interest in attempting to decrease the accumulation of intermediates (especially PAC and PAL) and to favour their conversion into VG. Subsequently, identification and overexpression of an eventual rate limiting enzyme from the vanillin biosynthetic pathway may serve to enhance conversion of pathway intermediates into the final product, VG. Furthermore, all the strains, including the reference, were found to have poor respiratory capacity and thereby high ethanol yield. Improving respiratory capacity of the selected overproducers will be an essential feature for future work. In fact, low dilution rate continuous cultivation (concurrent with better respiration) of an overproducer strain resulted in notably high titer of vanillin β-D-glucoside - 500 mg/l, 5-fold higher than the 45 mg/l reported by Hansen and co-workers [5].

In summary, the framework presented in this study comprises of i) *in silico *target prediction that accounts for the available physiological information; ii) systematic ranking of the targets based on the predicted metabolic adjustments; iii) *in vivo *verification through genetic engineering and fermentation; and iv) reaction/metabolite centric analysis of the experimental results. Our results demonstrate the applicability of *in silico *modelling tools for overproduction of a product from a multistep heterologous pathway in a eukaryotic system.

## Materials and methods

### Model simulations

Five new reactions were introduced in the *Saccharomyces cerevisiae *stoichiometric model [22] to convert 3-dehydroshikimate, a natural intermediate in aromatic amino acids biosynthesis, into vanillin β-D-glucoside (VG). Furthermore all the intermediates in the pathway were allowed to be secreted, based on the experimental evidence. FBA simulations were performed using linear programming library GLPK ftp://ftp.gnu.org/gnu/glpk/, while MOMA simulations were performed using quadratic programming library OOQP [45].

Strategy design to improve VG production in *S. cerevisiae *was performed by using OptGene with Biomass-Product Coupled Yield (BPCY) as design objective function [16].

### Minimization of metabolite turnover

Metabolite turnover or flux-sum is the sum of all incoming or outgoing fluxes around a particular metabolite under pseudo-steady state conditions [42,46]. Let Φ_i _denote metabolite turnover of metabolite i and its mathematical definition is given by $\Phi_{i}=\frac{1}{2}\sum_{k}\left|S_{ik}v_{k}\right|$, where *S_ik _*represents the stoichiometric coefficient of metabolite *i *in reaction *k *and *v_k _*is the flux of reaction *k*. By calculating the sum of all absolute flux values through (in and out) a given metabolite eliminates further concern about reactions reversibility. Furthermore, given the steady state assumption, the metabolite turnover will be half of the calculated sum.

Due to existence of alternative optimal FBA solutions within genome-scale models, *minimum of metabolite turnover *was calculated, given predetermined exchange fluxes (including growth), using a linear programming formulation as follows:

$$\begin{array}{l} \text{min}\Phi_{i}\\ s.t.\\ \sum_{j}S_{ij}v_{j}=0\\ \alpha_{j}\leq v_{j}\leq \beta_{j}\text{ for }\alpha_{j}\text{ and }\beta_{j}\in ℜ\text{ and }\alpha_{j}\leq \beta_{j}\text{ (Flux capacity constraint s, including uptake and secretion reactions)} \end{array}$$

### Plasmids and strains

The strain *Saccharomyces cerevisiae *VAN286 obtained by Hansen *et al. *was used as the background strain in this work. In order to produce vanillin, this strain must be transformed with a plasmid containing the gene *EntD *from *Escherichia coli*, coding for a PPTase. This enzyme is indispensable for post-translational activation by phosphopantetheinylation of ACAR in *S. cerevisiae *[5]. All the strains constructed during this study (Table 3) were transformed with the plasmid containing *EntD *prior to cultivation in order to produce VG.

**Table 3:** Yeast strains and plasmids used in this study.

Yeast Strain	Relevant genotype			Reference
VAN286	*MAT*a *his3*D1 *leu2*D0 *met15*D0 *ura3*D0 *adh6*::*LEU2 bgl1*::KanMX4 PTPI1::3DSD [AurC]::HsOMT [NatMX]::ACAR [HphMX]			Hansen *et al. *2009 [5]
VG0	*MAT*a *his3*D1 *leu2*D0 *met15*D0 *ura3*D0 *adh6*::*LEU2 bgl1*::KanMX4 PTPI1::3DSD [AurC]::HsOMT [NatMX]::ACAR [HphMX]::UGT72E2 [*HIS3*]			This study
VG1	*MAT*a *his3*D1 *leu2*D0 *met15*D0 *ura3*D0 *adh6*::*LEU2 bgl1*::KanMX4 PTPI1::3DSD [AurC]::HsOMT [NatMX]::ACAR [HphMX]::UGT72E2 [*HIS3*] *gdh1*			This study
VG2	*MAT*a *his3*D1 *leu2*D0 *met15*D0 *ura3*D0 *adh6*::*LEU2 bgl1*::KanMX4 PTPI1::3DSD [AurC]::HsOMT [NatMX]::ACAR [HphMX]::UGT72E2 [*HIS3*] *pdc1*			This study
VG3	*MAT*a *his3*D1 *leu2*D0 *met15*D0 *ura3*D0 *adh6*::*LEU2 bgl1*::KanMX4 PTPI1::3DSD [AurC]::HsOMT [NatMX]::ACAR [HphMX]::UGT72E2 [*HIS3*] *pdc1 gdh11*			This study
VG4	*MAT*a *his3*D1 *leu2*D0 *met15*D0 *ura3*D0 *adh6*::*LEU2 bgl1*::KanMX4 P_TPI1_::3DSD [AurC]::HsOMT [NatMX]::ACAR [HphMX]::UGT72E2 [*HIS3*] *pdc1 gdh1 GDH2*::*P*_PGK1_-*GDH2*			This study

**Plasmid**	**Gene content**	**Plasmid type**	**Selection marker**	**Reference**

pJH589	*EndD *(*E. coli*)	*CEN*-ARS (*S. cerevisiae*)	*URA3*	Hansen *et al*. [5]
pJH665	*UGT72E2 (Arabidopsis thaliana)*	*CEN*-ARS (*S. cerevisiae*)	*URA3*	Hansen *et al*. [5]
pARB021	*UGT72E2 (Arabidopsis thaliana)*	Integration (*S. cerevisiae*)	*HIS3*	This work
pWJ1042	Recyclable *URA3 (Kluyveromyces lactis)*	*CEN*-ARS (*S. cerevisiae*)		Reid *et al. *[51]
pPGK1-GDH2	P_PGK1_-*GDH2 *(*S. cerevisiae*)	Integration (*S. cerevisiae*)	*KanMX3*	Nissen *et al*. [8]

#### Cloning UGT72E2 in S. cerevisiae VAN286

The integrative plasmid pARB021 containing the gene UGT72E2 from *Arabidopsis thaliana *coding for a UDPG-glucosyltransferase was obtained by replacing the *URA3 *marker by the *HIS3 *marker in plasmid pJH665 with the restriction enzyme *XmaI*. Restriction enzymes and buffers from New England Biolabs were used and the conditions for restriction followed manufacture instructions. The *HIS3 *gene was PCR amplified using the primers His3_Fw and His3_Rev (Additional file 1, Table S1), the plasmid pWJ1213 [47] was used as template DNA and amplification was achieved using Phusion™ Hot Start High-Fidelity DNA Polymerase (Finnzymes Oy, Espoo, Finland). GFX™ PCR DNA and Gel Band Purification Kit (GE Healthcare) were used for DNA purifications and ligation was performed with T4 DNA ligase (New England Biolabs). The plasmid was treated with Antarctic Phosphate (New England Biolabs) in order to avoid recirculation. The ligation mixture was deactivated and transformed into chemo-competent *DH5α E. coli *cells. Ampicillin resistance was used as *E. coli *selection marker and plasmid extraction was performed using a GenElute HP Plamsid Miniprep Kit (Sigma-Aldrich). The plasmid ARB021 was verified by restriction analysis and sequencing of the PCR amplified *HIS3 *marker with the primers MarkSeq_Fw and MarkSeq_Rev from Additional file 1, Table S1 (StarSEQ, Mainz, Germany). The correct plasmid was then restricted with *SphI *and transformed into the yeast *TPI1 *promoter locus of VAN286, thus creating the strain VG0, producing VG from glucose. High efficiency yeast transformation method was used to construct the yeast strains [48].

#### Model guided strain construction

*PDC1 *and *GDH1 *gene deletions, as well as *GDH2 *overexpression were achieved by gene targeting through homologous recombination of bipartite PCR fragments, using *URA3 *gene from *Kluyveromyces lactis *as a marker [49]. The marker was flanked by direct repeats that allowed restoring of uracil auxotrophy by plating the cells in agar medium containing 5-Fluoroorotic acid (5-FOA) after each genetic manipulation [50].

The primers used for amplifying the up and downstream regions flanking the *PDC1 *and *GDH1 *gene (approximately 500 bp each) are listed in Additional file 1, Table S1, as well as the primers used to amplify *K. lactis URA3 *flanked by direct repeats from the plasmid pWJ1042 [51]. Strain VG1 was obtained by deleting the gene *GDH1 *in the strain VG0. Strain VG2 was obtained by deleting the gene *PDC1 *also in the strain VG0. Strain VG3 was obtained by deleting gene *GDH1 *in the strain VG2. The deletions were verified by analytical PCR using the primers PDC1_Ver_FW and PDC1_Ver_REV for *PDC1 *deletion, and GDH1_Ver_FW and GDH1_Ver_REV for *GDH1 *deletion (Additional file 1, Table S1). Strain VG4 was obtained from VG3 by swapping the native *GDH2 *promoter by the strong constitutive promoter of the gene *PGK1*, as previously reported by Nissen *et al*. [8]. A 500 bp fragment upstream the *GDH2 *open reading frame (ORF) used for homologous recombination was obtained from VG3 genomic DNA with the primers GDH2(UP)_Fw and GDH2(UP)_Rev (Additional file 1, Table S1). The downstream fragment used for homologous recombination was amplified from the plasmid pPGK1-GDH2 [8] with the primers PGK1_GDH2(Dw)_Fw and PGK1_GDH2(Dw)_Rev listed in Additional file 1, Table S1. 1479 bp of the *PGK1 *promoter region were used to substitute the *GDH2 *original promoter, while the initial 500 bp of the *GDH2 *OFR were used to ensure accurate targeting. The promoter swapping was verified by analytical PCR with the primers PGK1verif and Gdh2verif (Additional file 1, Table S1), amplifying 420 bp of the *PGK1 *promoter to 1300 bp of *GDH2*.

### Medium Composition

A defined minimal medium as described by Verduyn *et al. *(1992) with 20 g/l glucose as sole carbon source was used for cell cultivation [52]. The medium composition used for batch and continuous cultivation in well controlled bioreactors was as follows: 5.0 g/l (NH_4_)_2_SO_4_, 3.0 g/l KH_2_PO_4_, 0.5 g/l Mg_2_SO_4_, 2.0 ml/l trace metal solution, 1.0 ml/l vitamins solution, 0.05 ml/l antifoam 204 (Sigma-Aldrich A-8311) and 80 mg/l L-methionine. Trace metal solution contained 3 g/L FeSO_4_.7H_2_O, 4.5 g/L ZnSO_4_.7H_2_O, 4.5 g/L CaCl_2_.6H_2_O, 0.84 g/L MnCl_2_.2H_2_O, 0.3 g/L CoCl_2_.6H_2_O, 0.3 g/L CuSO_4_.5H_2_O, 0.4 g/L NaMoO_4_.2H_2_O, 1 g/L H_3_BO_3_, 0.1 g/L KI and 15 g/L Na_2_EDTA.2H_2_O. Vitamins solution included 50 mg/l d-biotin, 200 mg/l *para*-amino benzoic acid, 1.0 g/l nicotinic acid, 1.0 g/l Ca-pantothenate, 1.0 g/l pyridoxine HCL, 1.0 g/l thiamine HCl and 25 mg/l m-inositol.

The pH was adjusted to 5 by addition of 2N NaOH prior to autoclavation, the glucose was autoclaved separately and methionine and vitamins solutions were sterile filtered (0.2 μm pore-size Ministart^®^-Plus Sartorius AG, Geottingen, Germany) and added after autoclavation.

### Batch cultivations

Batch cultivations were executed in well-controlled, aerobic, 2.2 B Braun Biotech Biostat B fermentation systems with a working volume of 2 L (Sartorius AG, Geottingen, Germany). Proper mixing conditions were ensured by two disk-turbine impellers rotating at 800 RPM and 4 baffles. The pH was automatically controlled at 5 by addition of 2N NaOH. The temperature was kept constant at 30°C. The air flow rate was 1 vvm (volume air per volume of broth per minute).

Prior to inoculation, 100 ml precultures were cultivated in 500 ml baffled shake-flasks at 30°C until OD_600 nm _5 in an orbital shaker (150 RPM). Minimal medium as described above was used to grow the precultures with 20 g/l glucose. The bioreactors were inoculated to an initial OD_600 nm _ranging from 0.5 to 0.7.

### Continuous cultivations

Aerobic, carbon limited continuous cultivations were carried out in 2.2 B Braun Biotech Biostat B fermentation systems (as described above for batch cultivations) with a constant working volume of 1.5 L. The temperature was kept at 30°C, the pH was maintained at 5 by addition of 2N NaOH, the stirring speed was 600 RPM and the air flow was 1 vvm. Minimal medium with 20 g/l glucose was used to feed the bioreactors at a constant dilution rate of 0.1 h^-1^. The volume was kept constant at 1.5 l by controlling the level of broth inside the vessel. Steady state conditions were assumed after at least 5 residence times and CO_2 _and biomass concentrations were constant.

### Off-gas analysis

For both cultivation modes (batch and continuous), off-gas passed through a condenser to minimize evaporation loss during the fermentation and filter sterilized before carbon dioxide and oxygen were quantified in a Brüel & Kjær 1308 acoustic gas analyser (Brüel & Kjær, Nærum, Denmark).

### Biomass determination

Samples were maintained at 4°C post sampling and the biomass concentration was monitored by optical density at 600 nm (OD_600 nm_) and dry cell weight. OD_600 nm _was measured throughout all the fermentation in a Shimadzu UV mini 1240 spectrophotometer (Shimadzu Europe GmbH, Duidberg, Germany). The samples were diluted with distilled water in order to obtain measurements in the linear range of 0 to 0.6 OD_600 nm_. Dry cell weight was determined by filtering a known volume of fermentation broth with pre-dried 0.45 μm pore-size nitrocellulose filters (Sartorius AG, Geottingen, Germany), which were subsequently washed with a 3× sample volume 0.9% NaCl saline solution. The filters were then dried for 20 minutes at 150 W in a microwave oven and kept in a desiccator while cooling for at least 2 h. The filters where finally weighted using an analytical balance.

### Glucose and external metabolites analysis

The fermentation samples were immediately filtered using a 0.45 μm pore-size syringe-filter (Sartorius AG, Geottingen, Germany) and stored at -20°C until further analysis. Glucose, ethanol, glycerol, pyruvate, succinate and acetate were determined by high performance liquid chromatography (HPLC) analysis using an Aminex HPX-87H ion-exclusion column (Bio-Rad Laboratories, Hercules, CA). The column temperature was kept at 60°C and the elution was performed using 5 mM H_2_SO_4 _with flow rate of 0.6 ml/min. Metabolites detection was performed by a RI-101 differential refractometer detector (Shodex) and an UVD340U absorbance detector (Dionex) set at 210 nm.

Extracellular vanillin, vanillin β-D-glucoside (VG), protocatechuic acid (PAC), protocatechuic aldehyde (PAL) and vanillic acid were quantified by high performance liquid chromatography (HPLC) using Agilent 1100 series equipment with a Luna C18 column (Phenomenex). A gradient of methanol (+ 1% tetra-fluoroacetic acid) and water (+ 1% tetra-fluoroacetic acid) at a flow rate of 0.3 ml/min was used as mobile phase. The column was kept at 300 bar and 30°C. Metabolite detection was performed using a UV diode-array detector set at 280 and 310 nm.

## Competing interests

The authors declare that they have no competing interests.

## Authors' contributions

KRP conceived the study. ARB, KRP and UM designed the study. ARB and CM performed the experiments. ARB analyzed the data and drafted the manuscript. ARB, KRP, UM, JH and BLM contributed to the analysis of the results and manuscript writing. KRP, UM, JH and BLM supervised and co-ordinated the overall study. All authors read and approved the final manuscript.

## Supplementary Material

Additional File 1 **Table S1: List of primers used in this study**. Click here for file
