# Supplementary material for: Improved vanillin production in baker's yeast through in silico design
Source: Microb Cell Fact. 2010 Nov 8;9:84. doi: 10.1186/1475-2859-9-84 (PMC2992047; doi:10.1186/1475-2859-9-84)
Supplement: Additional File 1 — Table S1: List of primers used in this study. [file 1475-2859-9-84-S1.DOC]

Table S1: List of primers used in this study.

| **Primer name** | **Primer sequence** | **Purpose** |
| --- | --- | --- |
| His3_Fw | attgctatgacccgggCATAACACAGTCCTTTCCCGC | Amplify *HIS3* marker from pWJ1213 |
| His3_Rev | attgctatgtcccgggTCACACCGCATAGATCCGTC | Amplify *HIS3* marker from pWJ1213 |
| MarkSeq_Fw | CTGTGTCTATGAAAGTCGACGCG | Sequencing marker in plasmid ARB021 |
| MarkSeq_Rev | GGGGCTGGCTTAACTATGC | Sequencing marker in plasmid ARB021 |
| dKL5’ | gtcagcggccgcatccctgcTTCGGCTTCATGGCAATTCCCG | Amplify N-terminal URA3 from plasmid pWJ1042 |
| Kl URA3 3’-int | GAGCAATGAACCCAATAACGAAATC | Amplify N-terminal URA3 from plasmid pWJ1042 |
| Kl URA3 5’-int | CTTGACGTTCGTTCGACTGATGAGC | Amplify C-terminal URA3 from plasmid pWJ1042 |
| cKL3’ | cacggcgcgcctagcagcGGTAACGCCAGGGTTTTCCCAGTCAC | Amplify C-terminal URA3 from plasmid pWJ1042 |
| GDH1(UP)_Fw | GTCATCATTTCAAATATATG | Amplify *GDH1* upstream fragment from VG0 genomic DNA |
| GDH1(UP)_Rev | gcagggatgcggccgctgacATAGTCTAAAAGAAAGAAAA | Amplify *GDH1* upstream fragment from VG0 genomic DNA |
| GDH1(DW)_Fw | ccgctgctaggcgcgccgtgTTCTTTTTCTTTTTGGTCTC | Amplify *GDH1* downstream fragment from VG0 genomic DNA |
| GDH1(DW)_Rev | AAAGTATACGTAATCTAAGT | Amplify *GDH1* downstream fragment from VG0 genomic DNA |
| GDH1_Ver_FW | TTGCAAGTTAAAGCGGTC | Analytical PCR for verifying *GDH1* deletion from VG2 genomic DNA |
| GDH1_Ver_REV | GCCCATGCATTTTCAGT | Analytical PCR for verifying *GDH1* deletion from VG2 genomic DNA |
| PDC1(UP)_Fw | TCGTTTAAGAGAAATTCTCC | Amplify *PDC1* upstream fragment from VG0 genomic DNA |
| PDC1(UP)_Rev | gcagggatgcggccgctgacGCGATTTAATCTCTAATTAT | Amplify *PDC1* upstream fragment from VG0 genomic DNA |
| PDC1(DW)_Fw | ccgctgctaggcgcgccgtgTTTGATTGATTTGACTGTGT | Amplify *PDC1* downstream fragment from VG0 genomic DNA |
| PDC1(DW)_Rev | GTGATGGCACATTTTTGCAT | Amplify *PDC1* downstream fragment from VG0 genomic DNA |
| PDC1_Ver_FW | AGCAATGGCTTGCTTAATAG | Analytical PCR for verifying *PDC1* deletion from VG2 genomic DNA |
| PDC1_Ver_REV | ATTTGCAAAATGCATAACCT | Analytical PCR for verifying *PDC1* deletion from VG2 genomic DNA |
| GDH2(UP)_Fw | AGCAATGTCATACTGGCC | Amplify *GDH2* upstream fragment from VG3 genomic DNA |
| GDH2(UP)_Rev | gcagggatgcggccgctgacTTGAGATCGTGACAATCAC | Amplify *GDH2* upstream fragment from VG3 genomic DNA |
| PGK1_GDH2(Dw)_Fw | ccgctgctaggcgcgccgtgTCTAACTGATCTATCCAAAACTG | Amplify *PGK1_GDH2* (DW) fragment from pPGK1_GDH2 |
| PGK1_GDH2 (DW)_Rev | GAATCATCCATTTCAATCC | Amplify *PGK1_GDH2* (DW) fragment from pPGK1_GDH2 |
| PGK1verifb | GTCACACAACAAGGTCCTA | Analytical PCR for verifying GDH2 overexpression from VG4 genomic DNA |
| Gdh2verifb | GGTTTTCTACAATCTCCAAAAGAG | Analytical PCR for verifying GDH2 overexpression from VG4 genomic DNA |

^a^Small case letters indicate non homologous region of the primer, rather they code for restriction sites for cloning purposes or tails for fusion PCR.

^b^Primers by TL Nissen and co-workers [8].
